# Inaugural Dissertation on the Presence of Air in the Organs of Circulation

**Published:** 1838-10

**Authors:** 


### Art. XII.

1. *Inaugural Dissertation on the Presence of Air in the Organs of Circulation; submitted to the Medical Faculty of the University of Edinburgh, &c.* By John Rose Cormack, President of the Royal Medical Society of Edinburgh, &c.—*Edinburgh*, 1837. 8vo. pp. 56.

2. *Remarks on a Case of Suicide, published by* Dr. P. D. Handyside, *in No.* cxxxiv. *of the Edinburgh Medical and Surgical Journal.* By John Rose Cormack, m.d.—*Edinburgh*, 1838. 8vo. pp. 15.

3. *Bulletin de l'Académie Royale de Médecine.* Tome II. No. 12. *Rapport de* M. BOUILLAUD *sur les Expériences relatives à l'Introduction de l Air dans les Veines, faites par* M. AMUSSAT.—*Discussion sur l'Introduction de l'Air dans les Veines. Ib.*

4. *Lettre sur l'Introduction de l'Air dans les Veines de l'Homme.* Par M. VELPEAU. *Gazette Médicale,* 29 *Fev.* 1838.

THE question of introduction of air into the veins is not less interesting to the practical surgeon than to the physiologist. If any proof were required of the interest which this subject is calculated to excite, it might be found in the appointment, by the Royal Academy of Medicine of Paris, of a Commission of Inquiry; in the report of the commissioners; and in the protracted discussion to which that report has given rise. In the present article we propose giving a careful analysis of the labours of the commission, and of the facts advanced during the discussion; we shall also examine the paper of M. Velpeau, as well as a case recently published by Dr. Handyside, with Dr. Cormack's remarks upon it, and the prize essay of Dr. Cormack. As some arrangement is necessary in order to place the several facts derived from these sources in their proper light, we shall, in the first place, analyze the principal experiments which have been made on animals, and then proceed to detail the cases in which air has been introduced into the veins of the human body.

I. The forcible introduction of air into the veins has been long practised by veterinary surgeons, as a prompt and certain means of destroying life; and the experiments made by physiologists previously to the labours of the Commission have also consisted in the forcible introduction of air, either by means of a syringe or from the mouth of the operator. In the experiments made by Nysten, in 1811, and cited at length in the Report of the Commission, air was injected by means of a syringe; in those of Cormack, it was blown through a tube. We shall, in the first place, state the principal results of these experiments as briefly as possible.

In four experiments made by Nysten, a quantity of air, varying from thirty-five to fifty-one cubic inches (English), was injected into the jugular vein of spaniels, in separate quantities, varying from eight and a half to eleven cubic inches, in a period of from five and a half to twelve minutes. Death followed almost immediately on the last injection of air. In one instance, death was instantaneous; in two instances, it occurred at the end of two minutes; and in the fourth case, though the exact time is not stated, it was evidently very short. In the fifth experiment, thirty-seven and a half cubic inches of air were injected at once into the jugular vein of a small dog: death ensued in a few seconds. Cormack relates three experiments, in which death followed promptly on the forcible injection of air into the veins.

*Experiment* 1. Horse, seventeen hands high. Two full inspirations blown into the left jugular vein, through a tube a quarter of an inch in diameter. Duration of the experiment, one minute. Death in three minutes from the commencement of the experiment. Quantity of blood lost not greater than in an ordinary venesection.

*Experiment* 2. Rabbit. Three or four full inspirations introduced into the right jugular vein, through a tube the size of a crowquill, in the space of two minutes. Death in a few seconds after the termination of the experiment.

*Experiment* 3. Dog. A large quantity of air introduced suddenly, and with great force, into the jugular vein, in the space of six seconds. Death in six seconds from the end of the experiment.

From these experiments of Nysten and Cormack, it appears:

1. That considerable quantities of air may be injected into the veins, without proving instantly fatal.

2. That death follows the introduction of air more promptly as the quantity injected at one time, and the force employed in injecting it, are greater.

3. That the sudden and forcible introduction of a large quantity of air proves almost instantaneously fatal. (Experiment 5, Nysten. Experiment 3, Cormack.)

With regard to the quantity of air necessary to cause death, the report of the Commission has the following note:

"Each time that Nysten injected a large quantity of air into the veins of animals with one stroke of the piston, death took place in the same manner as in the fifth experiment. When the animals were small and weak, as was the case with some spaniels, forty to fifty cubic centimètres (about fifteen and twenty cubic inches English) sufficed to cause almost instantaneous death. When large dogs, above the average size, were submitted to experiment, it was necessary to inject at once 100 to 120 cubic centimètres, (about forty and forty-eight cubic inches English.) Quantities much more considerable were required to destroy larger animals, such as oxen and horses, which are frequently killed in this way by veterinary surgeons."

The effects produced by the injection of air were not always equally marked. Thus, in the second experiment of Nysten, eight cubic inches of air, injected five times in succession, at intervals of less than two minutes, produced no severe symptoms; and a further introduction of twelve cubic inches did not cause instantaneous death. In the first experiment, on the contrary, in which the same quantity of air was injected into the jugular vein of an animal nearly twice the size of the one employed in the third experiment, the first injection of eight cubic inches of air was immediately followed by acceleration of pulse, and *a sound was heard analogous to that which is made by beating together white of egg and water: this sound came from the region of the heart, was synchronous with the systole of that organ, and depended on the mixture of the blood contained in the right cavities of the heart with the air injected.* It ceased entirely at the end of two minutes, and the pulse regained the frequency which it had before the experiment. A further injection of ten cubic inches of air was thrice repeated, and the animal died almost immediately after the last injection.

The symptoms produced by the injection of air into the veins vary in degree and in kind, in different experiments. Cormack briefly describes them thus: "The animal suddenly falls down, utters some cries, and speedily expires in convulsions." The symptoms observed in the experiments which have been cited are the following: Cries; frequent and violent efforts to inspire; feeble, fluttering, and often imperceptible action of the heart; convulsions; tetanic spasms; involuntary ejection of the urine and fæces; a sound resembling the beating up of white of egg and water, coming from the region of the heart, and synchronous with the systole of its ventricles.

Though these symptoms occurred in one or more of the instances alluded to, they are by no means constant. The tetanic spasms, and the involuntary discharge of urine and fæces, are mentioned only in the fifth experiment of Nysten; whilst the peculiar sound coming from the region of the heart was observed only in his first experiment. The effects produced by the sudden injection of thirty-seven and a half cubic inches of air are thus described: "After some seconds, cries, imperceptible pulse, violent agitations, opisthotonos, ejection of urine and fæces, some deep inspirations, and death."

*The post-mortem appearances* are not minutely detailed in the experiments of Nysten. The right cavities of the heart are stated to have been greatly distended by a mixture of air and frothy blood, while the left were entirely free from air. In the experiments of Cormack the air seems to have been more carefully traced through the circulating organs; it was not confined to the right side of the heart, but found its way in smaller quantity into the left; it existed in considerable abundance in the large veins near the heart, and in those of the abomen; and in one instance (Exp. 1) "air was found in every visible vein over the whole body." In the second and third experiment, no air was found in the left cavities of the heart; in the first experiment, however, "the left auricle contained frothy blood, and some coagulated masses. In the left ventricle the quantity of air was just sufficient to make its existence appreciable; but there was a great quantity of blood both fluid and coagulated in this cavity." (p. 11.) In the third experiment air was found in the coronary veins, and it is stated "as a circumstance worthy of note, that there was but little frothy blood in the pulmonary artery." No emphysema of the lungs existed in any of Cormack's experiments, and in the only one of the five experiments of Nysten in which the state of the lungs is noticed, emphysema was also absent. In detailing the post-mortem appearances in the third experiment, Cormack, after alluding to the distention of the right cavities of the heart, vena cava, abdominal veins, and those of the lower extremity, and remarking the absence of air from the left cavities of the heart and from the pulmonary arteries, says, "The obvious explanation of these phenomena is, that the air had been sent directly through the right auricle into the inferior vena cava and coronary veins; for its absence from the left side of the heart clearly indicates that what was found in the veins had not made the round of the circulation." The post-mortem appearances in the subject of the first experiment, seem to indicate the possibility of the air going the whole round of the circulation, although it finds its way into the left cavities of the heart in small quantities. Great venous congestion is a constant appearance.

In the experiments which have been quoted, the forcible injection of air into the veins caused death. We have still to notice those in which either no fatal consequences followed, or the animal survived the experiment some hours or even days. Two such experiments of Nysten may be briefly stated as follows:

*Experiment* 1. About seventy cubic inches of air injected into the jugular vein of a dog, in separate quantities of eight or twelve cubic inches. Eight injections were made occupying sixteen minutes, with intervals of two or three minutes between each two injections. The first injections caused a slight increase in the frequency of the pulse, without signs of pain. An injection of twelve cubic inches of air caused a slight increase in the respiratory movements. At the end of half an hour the animal presented no symptoms of disease; on the following day it ate its food as usual, and continued to enjoy perfect health till the tenth day from the experiment.

*Experiment* 2. One hundred cubic inches of air, injected in separate quantities at eleven times during the space of twenty-eight minutes, produced increased frequency of the pulse and respiration. When loosened after the last injection of air the animal lay on its side breathing naturally, and only appearing somewhat debilitated. An hour after the last injection a cough came on, and was renewed at intervals. The following day the cough was augmented, the breathing became laborious, and the pulse feeble. The dog was stretched out on the ground, and a transparent frothy liquid flowed from its mouth. Death thirty-five hours seven minutes after the last injection of air. Post-mortem inspection discovered the lungs gorged with blood, and with a large quantity of frothy mucus. Not a bubble of air was found in the heart or large vessels.

Nysten states that he repeated these two experiments frequently with the same results; that is to say, when he injected but a moderate quantity of air the animal soon recovered its health, but when he injected a large quantity of it, for instance, from eighty to one hundred cubic inches according to the size and strength of the animal, it was soon seized with an obstruction in the bronchi, producing cough, expectoration of frothy mucus, mucous rhonchus, and death. In some cases the animal did not die till the third day.

Three experiments of the same kind are related by Cormack. In one of these "*a large quantity* of air was injected into the jugular vein. Very violent effects were produced, so violent indeed, that the animal seemed for twenty minutes to be in a moribund, or at least in a very critical state. To our astonishment, however, it gradually came round, and in about an hour the inspiration, which was at first very laboured, became tolerably natural, and the rabbit was soon little out of sorts." This animal was killed after sixteen days; its lungs were quite healthy, and there was no air in any of the blood-vessels. In the two other experiments, "six or seven full expirations were injected through a small tube into the jugular veins of two dogs. A little difficulty of breathing ensued, but this soon passed away. At the end of half an hour the animals were killed, having up to that time seemed pretty lively, and exhibited almost no symptoms of uneasiness." On post-mortem inspection, lungs collapsed, no emphysema. "In both instances, when the scalpel was thrust into each of the cavities of the heart, a little air unmingled with blood rushed out with a whizzing noise."

In the two last experiments, the animals were killed so soon that no time was allowed to the lungs to become affected, as in the experiments of Nysten; the first experiment, however, more closely resembles those of Nysten and did not lead to the same results. It is impossible to establish an accurate comparison between the experiments of Nysten and Cormack, and we know not in which to place most confidence: Nysten's appear to us to have been made with the greatest care.

The experiments which we have just detailed, coupled with those first cited, establish the following results:

1. The forcible and sudden injection of considerable quantities of air into the veins proves almost instantly fatal.

2. The more gradual introduction of considerable quantities of air may either prove speedily fatal, (by embarrassing the action of the heart?) or cause death after an interval of many hours or even of a few days by exciting pneumonia, or may be productive merely of passing inconvenience.

3. When the injection of air proves *instantly or speedily fatal* the right cavities of the heart, and especially the right auricle, are found distended with air and frothy blood. Air is also to be found in all the large veins near the heart, particularly in the ascending cava and abdominal veins; it may also find its way into the veins of the extremities, and in rare instances is contained in every visible vein throughout the body. Air is rarely found in the left cavities of the heart, and then only in small quantities. When the injection of air has *not proved speedily fatal,* air is not to be found in any part of the circulating system.

4. Death, where it occurs soon after the injection of air, may be attributed to distension of the right cavities of the heart, and consequent impediment to the circulation: where death does not take place within some hours or days it may be referred to pneumonia.

These experiments clearly disprove the opinion of Bichât, that an extremely small quantity of air introduced into the veins is sufficient to cause death, as well as that of Leroy, that death is due in many instances to emphysema of the lungs.

Other experiments, both of Nysten and Cormack, still remain to be noticed, but these may be reserved till we come to discuss other questions connected with the introduction of air into the veins.

II. The researches of Nysten, in 1811, had no directly practical object, but were merely intended to solve a question of much interest to the physiologist. Cormack's treatise, published in 1837, throws little new light upon this subject, though it has now become as practically important as t was before physiologically interesting. Beauchêne, in the year 1818, first invited attention to the possible introduction of air into the veins during surgical operations; and, since his time, as many as thirty cases of real or supposed accidents of that kind have been published. In some of these cases the true cause of death is doubtful; and men of a sceptical turn of mind might impute the whole of them to other causes than the introduction of air into the veins. Hence a new class of experiments was obviously called for: the forcible injection of air into the veins could no longer serve any useful purpose beyond that of destroying life, and the duty of the experimenter was to teach the surgeon under what circumstances air *could introduce itself* into the veins; by what symptoms he might be warned of its admission; and to what remedial measures he ought to have recourse. These desiderata are supplied by the experiments of the commission appointed by the Royal Academy of Medicine.

Of these experiments, which are forty in number, twenty-nine were made on dogs, ten on horses, and one on a mule. The veins which were opened were the external jugular, subclavian, and axillary veins. In some instances these vessels were left in their natural position; in others they were put on the stretch by extension and separation of the limb. In some experiments the horizontal position was employed, in others the vertical; in some the animals were strong and healthy, in others purposely debilitated. The introduction of air was in some cases allowed to take place spontaneously; in others it was injected by a syringe or by the mouth.

We shall, on the present occasion, state as briefly as possible the chief results of these experiments, referring to the Third Part of this Number for an abridged abstract of the whole. We have thought it advisable to give this abstract, not merely because the experiments are in themselves interesting and valuable, but because, in consequence of the want of systematic method in their performance, it is almost necessary to examine them individually in order to appreciate the results deducible from the whole. In the following summary we shall first give the results of the forcible injection of air by a syringe or the mouth, and then examine the so-called spontaneous introduction of air.

A. As regards the forcible introduction of air the two following points seem clearly made out.

1. The injection of a considerable quantity of air is necessary to cause death or even to produce marked uneasiness.

2. The severity of the symptoms produced increases with the quantity of air injected, and with the force employed in its introduction.

It seems also probable, although not actually proved, that the injection of air which has been respired proves more speedily fatal than that of pure atmospheric air. As we have no means of instituting a strict comparison between the experiments performed with the two kinds of air, this statement must remain open to doubt. In two out of three experiments (20, 21, and 23 of the commission,) in which pure atmospheric air was injected into the jugular vein of dogs the animals recovered, whilst three experiments in which air was *gently* breathed into the vein through a tube, proved fatal. In these instances death took place in half a minute, one minute, and two minutes, respectively, from the first introduction of air. Three similar experiments made on horses proved fatal in five, five and a half, and six and a half minutes, respectively. In one of Cormack's experiments a dog was killed in twelve seconds, and in another a horse was destroyed in three minutes. Three other experiments, however, made by Cormack, prove that the insufflation of air is not necessarily fatal, for seven or eight inspirations did not destroy the life of two dogs, (they survived half an hour, and were then killed;) and a rabbit also escaped, though a large quantity had been thus introduced. Though it is highly probable, therefore, that the injection of respired air proves more fatal, and destroys life more speedily than that of pure atmospheric air, the confidence of the reporter of the commission (Bouillaud), as to this point, seems scarcely warranted by the experiments adduced. If the fact were fully established the explanation given by Bouillaud might be admitted as the true one, viz. that the more fatal effects of respired air are due to the change effected by respiration, and not in any degree to the mode in which the air is introduced. This point, it would seem, might be easily determined by injecting with a syringe a moderate quantity of atmospheric air having the same admixture of carbonic acid as it acquires in respiration. The cause of death, as well as some other questions suggested by the experiments in which air was forcibly injected, will be best discussed when we come to consider the so-called spontaneous introduction of air.

B. The experiments made to determine the conditions under which air may enter the veins, the symptoms produced by its admission, and the best means of remedying this accident, constitute the most original and valuable part of the labours of the commission. The practical bearing of these experiments will justify a more lengthened and careful analysis than we have thought it advisable to make of the experiments in which air was forcibly introduced. We propose to examine in succession each of the following points:

1. The conditions under which air may enter the veins.
2. The signs indicative of the admission of air.
3. The symptoms produced.
4. The period at which death takes place.
5. The post-mortem appearances.
6. The proximate cause of death.
7. The remedial measures which may be resorted to.
8. The application of these experiments made on animals, to the human subject.

1. *Conditions under which Air may enter the Veins.* In every instance in which an opening was made into that part of the external jugular vein in which the venous pulse, or the phenomenon of flux and reflux of blood, was observed, the introduction of air took place. When the opening was made into a part of the vein above the site of the venous pulse, no air was observed to enter (Exp. 1 and 30 of the commission); but if a tube was introduced into the opening and advanced as far as the site of the venous pulse (Exp. 19 and 29 of the commission), the entrance of air took place just as if the vein had been opened at its lower part. Air, however, may find admission into a vein in which no venous pulse exists, provided the orifice of the vein be kept open. Thus, in Expt. 9 of the commission, the axillary vein was opened where no venous pulse could be detected, and no introduction of air took place till the limb had been forcibly separated from the trunk, and the orifice of the vein kept on the stretch. In Experiment 10, however, the brachial vein having been opened at the lower edge of the axilla, and the admission of air favoured as much as possible, no air entered. Hence then, though air invariably enters a wound made into a vein in which the venous pulse exists, it *may* gain admission where the venous pulse is absent provided the orifice be kept open. The experiments do not determine the greatest distance from the part at which air may be introduced into a vein; but Experiments 9 and 10 of the commission, render it highly probable that, though it may find its way into the *axillary* vein when the position of the parts is favorable to its introduction, no means that can be resorted to will cause it to enter the commencement of the *brachial*. We may, therefore, fix upon some point between these two veins as the limit to the possible introduction of air; and as air did not enter the jugular when an opening was made into its upper part, or even into a point as low as the junction of the middle and lower third, we are justified in asserting that the limit of the possible admission of air into the veins is but little removed from the site of the venous pulse.

The following propositions embody the principal results obtained by the experiments of the commission under this head:

*a.* When an opening is made into a vein at any point at which the venous pulse exists, air immediately enters.

*b.* If an opening be made into a vein a short distance beyond the point at which the venous pulse exists, air may enter, provided the orifice be kept open; but if the opening be made at a considerable distance from the site of the venous pulse, no air enters, though the orifice be kept open and the admission of air be as much as possible encouraged.

The situation in which the introduction of air takes place points distinctly to the true cause of its admission. Barry and Poiseville have shown that during inspiration the blood of the large veins flows more freely towards the heart, and that during expiration it regurgitates. This effect does not extend, as Barry thought it did, to the small veins, but diminishes rapidly as we pass from the heart. Thus, in the experiments made by the commission, the venous pulse was observed in the lower third only of the external jugular vein; it was present also in the subclavian, but absent in the axillary and brachial veins. Now, wherever the venous pulse exists, wherever, in other words, the blood tends to flow more freely towards the heart with each inspiration, and to regurgitate with each expiration, then the contents of the vein, whether they consist of air or of blood, must be drawn into the heart at each inspiration, and again more or less completely expelled by each expiration. If an opening be made into a part of a vein somewhat beyond the situation of the venous pulse, and the aperture be kept open, air still gains admission; but if the aperture be not put on the stretch the pressure of the atmosphere approximates the sides of the vein, and the air does not enter. The introduction of a tube, however, by preventing the collapse of the vein, ensures the free admission of air. A rigid state of the coats of the vein would have the same effect.

2. *The Signs indicative of the Admission of Air into the Veins.* These consist of certain sounds heard in the vein itself and in the region of the heart. (*a.*) *In the vein* the sound resembles the *lapping* of a dog or cat, or, especially where the vein is very large, as in the horse, a loud *gurgling.* This sound is synchronous with the inspiration, but occasionally it is much more frequent, and then accompanies the diastole of the right cavities of the heart,—or of the right ventricle, according to Bouillaud, who states that the phenomena of flux and reflux have been seen to be synchronous not merely with the respiratory movements, but also with the diastole and systole of the right cavities of the heart. This, it may be remarked, harmonizes with the views of Mr. King on the safety-valve functions of the heart. (*b.*) *In the region of the heart*, as has been already remarked by Nysten, a *bruit de soufflet,* with or without a gurgling sound, is heard. This bruit continues for a long time after the air has ceased to gain admission into the vein, and is synchronous with the movements of the heart and not with those of respiration.

3. *The Symptoms produced.* At the end of from one to ten minutes from the first introduction of air, the respiration and circulation become embarrassed and frequent, the animal loses its strength, and is anxious and agitated. If the further introduction of air is prevented, the symptoms rapidly improve and the animal regains its strength and health. If, on the other hand, the air is still allowed to enter the vein, all the symptoms are aggravated, the respiration becomes more and more laborious, the debility is extreme, the urine and fæces are discharged involuntarily, cries, convulsions, and tetanic spasms occur, and death takes place in the majority of instances within half an hour of the introduction of air. If death takes place at a later period the animal seems to die of exhaustion.

4. *The Period at which Death takes place.* This depends on so many different circumstances that it will be difficult to come to a just conclusion respecting it. The age, size, and strength of the animals submitted to experiment will exercise a material influence. The report of the Commission directs attention to two points, the effect of position, and that of previous strength or debility. In the vertical position death takes place more speedily than in the horizontal. Thus in four experiments on dogs placed in the vertical position, death took place in three, six, sixteen, and twenty-five minutes, respectively; in four experiments, in which the horizontal posture was maintained death took place in four and a half, fifteen, twenty-five, and thirty minutes, respectively. The average of the first four experiments is twelve minutes and a half, that of the last four is eighteen minutes and a half. The extremes in the first case are three and twenty-five, in the last four and a half and thirty-six. Hence, then, death takes place more speedily when the animals are placed in the vertical position than when they are placed in the horizontal position. This statement is corroborated by the fact that, whenever the animals were held upright all the symptoms became more distressing, but were instantly relieved by the resumption of the horizontal position. The relief thus experienced seemed to depend on the escape of blood and froth from the orifice of the veins

The effect of debility in hastening the death of the animals submitted to experiment will be seen from the following comparison: Two dogs, not enfeebled before the experiment, (Expts. 3 and 5 of the Commission,) died, the one in a quarter of an hour, the other in half an hour: two others, purposely enfeebled before the experiment, (Expts. 6 and 7 of the Commission,) died in four and a half minutes and twenty-five minutes, respectively. Four horses, not debilitated before the experiment, (Exps. 30, 31, 32, 33, of the Commission,) died in fourteen, twenty-eight, thirty-five, and 104 minutes, respectively; whilst four other horses, debilitated before the experiment, (Expts. 34, 35, 36, 37, of the Commission,) died in nine, twelve, thirteen, and sixteen minutes, respectively. The mean of the first four experiments is forty-five minutes; the extremes, fourteen and 104; the mean of the last four experiments is twelve and a half, and the extremes nine and sixteen. Hence, death takes place more speedily in animals which have been purposely debilitated. The shortest period in which death took place in consequence of the spontaneous introduction of air was three minutes, in a dog placed in the vertical position, and not debilitated before the experiment; whilst four and a half minutes was the shortest time in a dog placed in the horizontal position, and debilitated before the experiment.

5. *Post-mortem Appearances.* (*a.*) The examination made immediately after death. The distension of the right cavities of the heart and of the pulmonary artery by frothy blood is a constant appearance, and sometimes increases the bulk of the organ to three times its natural extent. Sometimes (Expts. 6, 7 of the Commission,) unmixed air is found in those cavities. The left cavities of the heart, with one exception (Expt.

18), contained no air, and offered a striking contrast to the distension of the right side. In Expt. 18, only a few bubbles of air were discovered. The cranium having been opened in some dogs which had been placed in a vertical position, air-bubbles were found in the jugular of the opposite side, in the facial veins, and in the cerebral veins and sinuses. (Expts. 14, 22, 23, &c.)

(*b.*) Examination made some time after death. In one only out of four dogs, which were inspected from four to ten days after the introduction of air, (Expts. 12 and 13,) the heart and large vessels were found free from air. In this instance the heart was found collapsed and flabby. Air was detected either in the left cavities of the heart (Expt. 27,) or in the arteries and veins of the extremities, in each of the other three dogs. Hence, then, the air which is at first confined to the right side of the heart and pulmonary artery passes, at the end of a certain time, through the pulmonary veins to the left side of the heart, and thence to the arteries and veins of the whole body. In horses, in addition to the distension of the right side of the heart, the left cavities of that organ, the aorta, and the vessels of the brain, were found to contain air; and that in cases where the body was inspected directly after death. This difference is attributed, in the report of the Commission, and with apparent justice, to the larger size of the capillary vessels of the lungs, which admit of the more free passage of the air from one side of the heart to the other.

6. *The Proximate Cause of Death.* This, in the opinion of M. Bouillaud, is threefold: 1. The enormous distension of the right cavities of the heart by the air expanded by the heat of the blood, and the consequent impediment to the free contraction of that organ. 2. The presence of air in the pulmonary artery and its branches; which air, by rendering the blood spumous, prevents its free transmission through the lungs. 3. When the air enters the veins of the brain, the compression which it exercises on that organ. The first and third of these have been already mentioned by Nysten; but to the first alone is any importance attached by Cormack. "It may be stated," he says, "as clearly established, that when a quantity of air, by entering the circulation, proves suddenly fatal, the immediate cause of the arrestment of the vital functions is the inability of the right side of the heart to contract and expel its elastic contents; and therefore all the phenomena which follow are consequences of this first cause." (*Thesis*, p. 21.) The experiments of Dr. J. Reid on the paralysis which the right side of the heart undergoes when over-distended, (to which we have formerly referred when speaking of asphyxia,*) appear to us to confirm this view to a certain extent, by giving a reason for its inability to contract when air has been forcibly injected into a vessel leading to its cavity. With regard to the second cause adduced by Bouillaud, we are disposed to think that it has been too much overlooked. The degree in which its operation will be conceded, however, must depend upon the views entertained of the physiology of the circulation. If we believe (as we think we are not only warranted, but compelled to do,) that the changes which the blood undergoes in the

* See British and Foreign Med. Review, Vol. V. p. 104; and Edinburgh Med. and Surg. Journal, vol. xlv. p. 387.

capillaries, both of the lungs and of the general system, are necessary for the creation of a part of the motive powers of the circulation, it is obvious that, if these are stopped in any way, a check must also be put to the movement of the fluid. In asphyxia, we cannot doubt that the obstruction to the circulation commences in the capillaries of the lungs, in consequence of the deficient supply of oxygen preventing the due aeration of the blood; and that the paralysis of the right ventricle from over-distension, and that of the left from want of its required stimulus, are but results of this primary cause. Now, where death takes place rapidly from the forcible insufflation of air into the veins, it is easy to perceive how the distension of the right ventricle, and the consequent stoppage of the circulation, are the immediate results of the process. But where air has spontaneously entered, and is found to have passed into the pulmonary artery, it does not seem unreasonable to suppose that it may prevent the passage of the blood with which it is mixed through the lungs; the due relation between the blood in the vessels and the air in the cells being just as much disturbed by the admixture of air in the former as by its deficiency or impurity in the latter: and, if the blood be thus obstructed in the lungs, it is obvious that, as the right ventricle already contains an extraneous bulk of air, it must speedily acquire a fatal distension, from the return of the venous blood from the system. That the admission of air into the vessels of the lungs may act also more mechanically appears probable from an experiment of Dr. Cormack's. He threw a quantity of air into one of the mesenteric veins of a rabbit, and in eight minutes afterwards killed the animal. *The liver was in a state of almost complete anæmia*, and, upon slicing it, minute bubbles of air appeared at every point on the incised surfaces. There was no air apparent in the heart, nor in any part of the body except the liver. (p. 26.) Is it not highly probable that the admission of air into the vessels of the lungs would produce a similar state of anæmia in those organs, and, by preventing the return of a due supply of blood to the left side of the heart, produce syncope and death? The non-existence of air in the cerebral vessels, in more than one experiment, disproves the opinion of Bichât that death results in all cases from the effect of the air on the brain. The opinion, also, that where the animal survives the experiment during some time, and ultimately sinks, death is due to emphysema of the lungs, is refuted by the experiments of the Commission, no less than by those of Nysten and Cormack. Whether or not each of the causes assigned in the report of the Commission bears a part in producing death must remain a matter of doubt. The extreme distension of the right side of the heart seems alone sufficient to account for the fatal result in the majority of cases.

7. *Remedial Measures.* Upon this question, so important in a practical point of view, the experiments of the Commission throw but little new light. The compression of the chest and abdomen (Expt. 18) does not prevent the admission of air into the vein. How, indeed, should such compression prevent the introduction of air, unless at the same time it stop the respiration? For we have seen that wherever an opening is made into a vein in which the venous pulse exists,—in which, in other words, the contents of the vein are drawn into the heart by the expansion of the chest,—air must gain admission. Compression of the chest and abdomen, therefore, cannot prevent the entrance of air into the veins. Can compression of these parts remedy the ill effects which follow the introduction of air? The experiments of the Commission are neither numerous nor accurate enough to determine this question. The only experiment (Expt. 27) in which compression was tried is valueless. The air had only continued to find admission into the vein during a quarter of a minute: now, from the introduction of so small a quantity of air, death would in all probability never have happened, even had compression of the chest and abdomen not been resorted to. It is true that the compression caused a quantity of frothy blood to issue from the vein; it might therefore have proved useful. If compression were repeatedly exercised, and the aperture of the vein closed after each compression, there is little doubt that good effects would follow, and that life might in some cases be preserved; but the experiments of the Commission throw no light on the subject. Two experiments (28 and 29) were made with a view of ascertaining the effect of withdrawing the air by means of a syringe; but these, too, are inconclusive; and in one of them the syringe was out of order. In the first experiment, a mixture of pure blood and froth was extracted; but in the second pure blood only was withdrawn. In both instances the animal recovered. All that we are justified in saying of the two remedies,—compression of the chest and abstraction of air by means of a syringe,—is that they are likely to prove beneficial, but that the experiments of the Commission lend us no assistance in forming an opinion. The symptoms produced by the introduction of air were evidently relieved, in more than one instance, by allowing the free exit of blood from the opening of the vein, and aggravated by closing the orifice. In two of Nysten's experiments, also, life seems to have been saved by the free flow of blood, in the one case, from an accidental wound; in the other, from a free opening made intentionally into one of the large veins. Nysten states that the beneficial effect of bloodletting was established by repeated experiments. The attention of the Commission does not seem to have been directed to this subject.

We have now brought our review of the labours of the Commission to a close; and, though we feel under obligations to them for some useful additions to our knowledge, we cannot but regret that a valuable opportunity has been, in a great measure, lost of throwing light upon a subject of great practical importance. If our limits would permit, we might be tempted to hazard some remarks on the *art of experimenting;* an art which the Commission have not thought it necessary to study or acquire. It is quite possible to introduce into experiments on animals the same accuracy, the same strictness, which we employ in our experiments on dead matter. The admirable experimental researches of Edwards have furnished a model which others would do well to imitate: those of the Commission will supply us with examples of many of those faults against which the inexperienced observer requires to be cautioned.

III. We now pass on to the application of experiments made on animals to the human subject. We shall not stop to discuss the difficult question, whether or not the experiments made upon animals are strictly applicable to the human subject; but, assuming that they are so, we shall compare them with fatal accidents which are stated to have followed the admission of air into veins in the neighbourhood of the chest, wounded during operations. Between thirty and forty such accidents have been described with greater or less minuteness, but there are but few of which the accounts are not open to serious objections. We shall arrange them in four groups: 1, The facts which are narrated upon hearsay; 2, Cases in which death has not followed the supposed admission of air; 3, Fatal cases in which no examination was made after death; 4, Fatal cases examined after death.

1. *Cases stated upon Hearsay.* These are four in number, and are stated to have occurred in the practice of M. Graefe, Sir A. Cooper, Lodge, and Duportail: they do not merit the slightest attention.

2. *Cases not followed by Death.* These are fifteen in number, and occurred in the practice of Mott, Clémot, Barlow, Warren, Roux, Mirault, Rigaud, Delaporte, Dubourg, Malgaigne, Bégin, Toulmouche, Amussat, and Velpeau. We shall notice each of them briefly. Mott (*Journal of Surg. and Med. Science, &c.* Nov. 1828, p. 127,) extirpated a tumour from the region of the parotid gland: the facial vein was opened; a peculiar sound was heard; the patient uttered a cry of distress, and nearly fainted. There is nothing characteristic in these symptoms, and the vein opened was too remote from the thorax to admit of air. In M. Clémot's case, a tumour was dissected from the region of the axilla: a sound of *aspiration* was heard; the patient fainted, but soon recovered. Here also there is nothing characteristic of the introduction of air. The same may be said of a second case, in which the same sound was heard after cutting a small vein beneath the clavicle; but no fatal consequences ensued. In a case by Barlow, a transient fainting was attributed to the division of a varicose vein on the *cheek;* a part at which the introduction of air seems impossible. Two cases are narrated by Dr. Warren, in his work on Tumours: in one of these (that of W. Buril, p. 298,) a cancerous tumour was extracted from the neighbourhood of the parotid gland: on exposing the carotid artery, with a view of applying a ligature to it, a vein was opened, and a sound was heard resembling the passage of air-bubbles through water. The patient complained of being ill; symptoms of apoplexy supervened, which were relieved by opening the temporal artery. No further bad consequences followed. Here the only symptom which could indicate the admission of air was the sound referred to. M. Roux's case is not worth mention. Rigaud, (*Quelques Faits de Pratique Chirurg.* Paris, 1836,) in performing an operation on the subclavian artery, opened a vein, which he took for the external jugular: a peculiar sound, a sort of aspiration of air, was heard three several times; but no dangerous consequences followed. M. Delaporte, (*Bulletin de l'Académie*, tome i. p. 132,) during the extirpation of a tumour from the neck, was alarmed by a whistling sound and sudden syncope. No dangerous consequences followed. The external jugular was opened by M. Malgaigne (*Gaz. Méd.* 1836, p. 167,) and the same whistling sound was heard; followed, however, by no bad symptoms. M. Amussat's case (*Bull. de l'Acad.* t. i. p. 894,) resembles the two former, except that the sound supposed to characterize the admission of air was *jerking*, and as it were *zigzag.* Velpeau relates the following case, (*Bull. de l'Acad.* t. i. p. 896:) having wounded the internal jugular vein during an operation, a distinct whistling was heard, followed by a sort of bubbling sound at the bottom of the wound. The patient cried out that she was dying, and fainted. The vein having been secured by the finger, the operation was completed without further accident. M. Bégin's case (*Presse Méd.* p. 463,) resembles the above: he opened the internal jugular, and immediately heard a sort of *glou-glou*, followed by no bad consequences. M. Mirault, in dissecting a tumour from the right side of the neck, wounded the internal jugular: three distinct whistling sounds were heard, and tetanic spasms followed. The patient soon recovered, but died suddenly at the end of three hours. In a case stated by M. Toulmouche, (*Bull. de l'Acad.* t. ii. p. 146,) a similar sound was followed by fainting. The operation was performed on the breast, and the supposed introduction of air took place during a forcible separation of the arm from the trunk.

In one or two of the above observations, the vein which was opened was situated beyond the point at which, in the experiments made on animals, air was found to enter the vein. The effects produced are, therefore, in all probability, referrible to other causes than the introduction of air into the veins. In the other cases, the veins which were wounded were within the distance from the thorax at which air may find admission into a vein. In these instances no fatal effects ensued. The syncope which occurred may or may not have been caused by the introduction of air. The peculiar sound which is stated to have been heard is almost the only ground for attributing the symptoms to that cause. The admission of air is not *proved* by these cases; but, granting that air did enter the wounded veins, we have the satisfaction of knowing that it is frequently followed by no dangerous consequences.

3. *Fatal Cases not examined after Death.* These are five in number, and occurred to Warren, Barlow, Goulard, Klein, and Mangeis. Warren's case is given at full length in the British and Foreign Medical Review, for April, 1838, p. 420: it leaves much to desire, and does not carry very strong conviction to our minds. In separating a tumour from its connexion with the axillary vein, "a vein was divided, and a small quantity of venous blood discharged. Scarcely was this done when *the patient struggled, her complexion changed to a livid colour, and at the same instant a bubbling or gurgling noise, which had not been noticed before, was heard, though indistinctly;* but the place from which it issued was not visible, the surrounding skin and fat lying over it. On this, the axilla was immediately compressed. The patient became insensible, *breathing as in apoplexy.* The tumour was at once separated. The posture of the patient was changed, and stimulants were diligently administered; but the pulse became less distinct at every instant, the lividity of the cheeks increased, the body grew cold, and the respiration more and more feeble until it entirely ceased. The lungs were inflated as a last effort, but without avail." We confess that we are sceptical as to this case. An *indistinct* bubbling or gurgling noise is the only symptom which appears to us to be characteristic; and in an *indistinct* sound of this sort, occurring in the course of an operation, we place but little confidence. The struggles might or might not have been caused by the introduction of air; the other symptoms produced might as easily be attributed to apoplexy from determination of blood to the head. It is to be regretted that a post-mortem examination was not made. The case narrated by Klein (*Gräfe and Walther's Journal,*) is even less convincing than that of Warren: he extirpated the thyroid gland in the case of a deaf and dumb child, and death took place in less than a minute. This case occurred in 1814, but was not narrated till long after that period, when it was adduced in confirmation of Dupuytren's case. The rapidity with which death occurred seems to be the only ground for attributing the fatal result to the introduction of air: but this very circumstance renders the assigned cause doubtful; for, in experiments on animals, three minutes was the shortest space of time in which the introduction of air proved fatal. Goulard's case is not more satisfactory than that just mentioned. In the course of an operation he wounded a vein, which he supposed to be the axillary vein, but from which little blood flowed. The patient was seized with convulsions, and died. As the introduction of air is not the sole cause of convulsions, we can scarcely admit this case as an instance of death from that cause. (See *Gazette Méd., &c.* 1833.) Barlow's case is more satisfactory. He was removing a tumour from the neck of a woman, and, whilst dissecting the skin, he heard a whistling and gurgling sound. The patient died suddenly, *without sighs or convulsions.* Sighing and convulsions, though usually present in the experiments on animals, were not invariable symptoms: the absence of them, therefore, does not entirely exclude this case from the number of those in which death was caused by the introduction of air into the veins. Mangeis bled a woman in the arm: eight ounces of blood had scarcely flowed from the vein, when the patient uttered a cry and expired. This case, too, is mentioned as an instance of the fatal effects of the introduction of air. We need not remark that there is no foundation whatever for the opinion.

4. *Fatal Cases examined after Death.* These are seven in number, and rest on the authority of Piedagnel (Beauchêne), Dupuytren, Castara, Delpech, Roux, Ulrich, and Putegnet.

Beauchêne (*Piedagnel, Thèse,* No. 250, Paris, 1827,) was removing a large tumour attached to the right shoulder of a man, twenty-three years of age. But little blood had been lost, when, on separating the last adherent portion of the tumour, M. Piedagnel heard a sound resembling the entrance of air through a small aperture into the chest of a living animal. It was the opinion of all present that the pleura had been wounded. The patient exclaimed, "Mon sang tombe dans mon cœur! je suis mort!" He became pale, his head fell backwards, the eyes were fixed, and he could not distinguish objects. Respiration was easy but loud, and seemed to be performed chiefly by the left lung; the movements on the right side of the chest being very feeble. The pulse was extremely small, frequent, hard, and irregular. The whole body was covered with a cold sweat, and he had some convulsions. All restorative means failed, and the patient died a quarter of an hour after the fatal cut had been given. On inspecting the body eighteen hours after death, the right pleura was found not to have been opened, but the external jugular vein had been wounded: in fact, a portion of this vessel (half its caliber, and an inch in length,) had been cut out. The wound in the jugular terminated just as it joined the subclavian. *None of the cavities of the heart contained any blood. The walls of the right cavities were flabby, very thin, pale, and of a much greater caliber than those of the*

*opposite side.* The brain presented a gray appearance, and all its blood-vessels, of a size sufficient to be visible, were distended with air. The aorta, crural arteries, and inferior vena cava, contained air mixed with blood. (See Cormack, pp. 14, 15.) The empty and flabby state of the cavities of the heart is peculiar, and did not exist in any of the animals submitted to experiment. The existence of air in the vessels seems to put the question as to the cause of death beyond all doubt. In Dupuytren's case, death may be ascribed, with equal confidence, to the same cause. In removing a tumour from a girl's neck, "a sound similar to that heard in the last case led the operator to remark that, had he been cutting in the neighbourhood of the air-passages, he would have supposed that he had made an opening into some of them. No sooner had he said this, and at the same time given the last stroke of the knife which concluded the separation of the tumour, when the patient exclaimed 'I am dying!' was seized with a general trembling, and quickly expired." "The body was examined twenty-four hours after death, and *the right auricle was found distended with air.*" "The other cavities of the heart (which were healthy), the arteries and veins throughout the body, and the membranes of the brain, contained fluid blood mixed with air." (*Cormack*, pp. 15, 16.) M. Castara's case is thus stated by Velpeau: "In 1826, a surgeon of Luneville, M. Castara, (*Saucerotte, Thèses de Strasbourg*, Mars, 1828,) removing a tumour which occupied the fossa infra-spinalis of the right shoulder, heard all at once a sort of *glou-glou* at the bottom of the wound. The patient, aged twenty-one years, fainted and expired suddenly, without convulsive movements. Twenty-four hours after death, the right cavities of the heart were found filled with liquid blood, mixed with a large quantity of air-bubbles. There were some bubbles of air also in the left cavities of that organ. The entire venous system of the upper extremity, including the forearm, was also filled with air-bubbles. The only vein opened was a branch of the sub-scapular, and the opening was less than a line in diameter." Upon this case M. Velpeau makes the following remarks: "There are in this fact some very remarkable particulars. It is the only one at present on record which can be compared, as far as the state of the blood in the heart is concerned, to the experiments made on animals. But the patient died suddenly; a circumstance which has never been observed in direct experiments. So small an opening, and that made into a vein beyond the axillary, does not allow the entrance of air to a degree to endanger life either in dogs or horses. We cannot understand, moreover, the presence of air in the veins of the right forearm, when this gas was absent from the inferior vena cava and from the left upper extremity. Though this fact of M. Castara is one of the most convincing, it leaves still some doubt on the mind." Delpech, (*Mémorial des Hôpitaux du Midi, deuxième année*, pp. 231—654,) in extirpating the arm at the shoulder-joint, heard two *snuffing* sounds, followed by fainting and instant death. The hemorrhage had been inconsiderable. The body was placed under water, and a considerable quantity of air-bubbles was found in the right cavities of the heart. In M. Roux's case, (see *Gaz. Méd.* 1833, p. 498, and *Transact. Méd.* t. x. p. 75,) a *whistling* sound was heard, whilst he was removing a tumour from the region of the parotid gland. The patient immediately uttered a plaintive cry, and was strongly agitated. A fainting took place, which was not of long continuance; the patient went on well for some days, but died at the end of a week. Air-bubbles were found in all the vessels. Though the presence of air in the veins proves its admission during the operation, there is no proof that the fainting was due to that cause; and there is no reason to attribute the death of the patient to it. Ulrich relates a case (*Journ. des Conn. Med. Chir.* tom. xi. p. 91; or *Gaz. Méd. de Berlin,*) in which the internal jugular was opened during an operation. A *whistling* sound was heard, and death took place in a minute. The right auricle was distended by air, but contained no blood. Black and liquid blood, without air, in the right ventricle. The suddenness of the death in this case is worthy of remark: as we have before stated, death never took place in animals in less than three minutes. In another case, stated by M. Roux, *Journ. des Conn. Méd.-Chir.* t. iv. p. 108; or *Revue Méd.* 1836, t. ii. p. 417,) gas was found in the heart and vessels after death, from a severe operation performed on a man already much debilitated. The upper extremity was extracted at the shoulder-joint, in consequence of a severe burn followed by sloughing of the whole limb. The patient, previously so greatly debilitated, fainted, and could not be restored. Some persons who were present *thought* that they heard a sort of whistling sound. Putegnet mentions the following fact in his thesis, (*Thèse,* No. 156, p. 41, Paris, 1834:) A man who had had an apoplectic fit was immediately bled from the jugular: death took place suddenly, and air was found in the right auricle.

The brief and imperfect sketch which we have given of the above facts, while it will form a sort of index, may prove useful by directing attention to many parts of this interesting subject which still demand careful examination. Comparatively few of the instances adduced can be regarded as satisfactory cases of the introduction of air into the veins. The report of the Commission selects the cases of Beauchêne, Dupuytren, Delpech, Roux, and Amussat, as the most conclusive. Velpeau regards the admission of air as *probable* in the cases of Bégin, Malgaigne, Mirault, Warren, Barlow, Delaporte, in one of the two reported by M. Roux, and by M. Clemot, and in the case mentioned by himself. He says that the introduction of air is *extremely probable* in the cases of Delpech and Ulrich; *and almost certain* in those of Dupuytren, Castara, and Goulard. He adds, however, that no one of these facts can be strictly compared to the results of direct experiment, and that all of them fail in producing complete conviction. With this opinion of Velpeau our own entirely coincides. If the experiments of the Commission could be assumed as the standard of comparison, to which all the cases of introduction of air into the veins of the human subject might be referred, almost all the cases reported would be effectually excluded by a want of strict resemblance in one or more of their symptoms to those observed in the case of animals. Thus, all the cases of sudden death from the supposed introduction of air would be shown to be fabulous, by the fact that three minutes was the shortest time in which death took place in the experiments on animals. The absence of convulsions or of any other symptom observed in the animals submitted to experiment would throw a doubt upon many other cases. The absence of distension of the right cavities of the heart by frothy blood would effectually exclude others; the position of the wounded vein would add to the difficulty; till at last no single conclusive case would remain. But there is one appearance observed after death, which can leave no doubt whatever in the mind of the most sceptical,—viz. the presence of air in the several parts of the circulating system. It is true that air may be *generated** in the vessels of the living body; but it is an extremely rare occurrence, and would be a singular coincidence indeed if it occurred in every person submitted to an operation. These cases, then, in which air was found in the circulating system after death, may be admitted as conclusive of the introduction of air through a wounded vein; but they cannot be regarded as satisfactory evidence that death was attributable to the presence of air: for we have seen that the admission of air into the veins is not necessarily attended by fatal consequences, and that a very considerable quantity of air may be introduced without causing even marked uneasiness. In the course of surgical operations, too, so many other causes exist capable of destroying life,—as loss of blood, exhaustion from excess of pain, fear or other strong emotion, determination of blood to the head, (as in one, if not in both the cases reported by Warren,)—that we ought to hesitate before we attribute the fatal consequences to the admission of air, even should air be discovered in the several parts of the circulating system. The tendency of the human mind to refer results to causes which are least understood, and most calculated to indulge the love of the marvellous, is well known: against this the medical man has especial need of being placed on his guard; and, in the case of the subject which we are now discussing, it is more particularly necessary. A better instance of the tendency to which we allude can scarcely be imagined than the one with which Dr. Handyside† has furnished us. A Mr. Doherty, who had been for some days labouring under delirium tremens, cuts his throat, and loses (according to Dr. H.'s own statement) a pound and a half of blood from a large number of divided vessels. Now, one would think that the sudden loss of this quantity of blood would be sufficient to cause and to explain death; but air was found in several arteries and veins:—*ergo*, death was attributable to the admission of air into the organs of circulation. But this appears to us, as well as to Dr. Cormack, a very hasty conclusion; and, with the view of showing our readers that it was so, we shall enter a little more fully into the details of the case, which are interesting in several points of view.

In the first place, we happen to know that the individual concerned (who was in the first year of his medical studies) had attended on the previous day a lecture on the anatomy of the neck; and the character of the incisions, which were not made in the usual situation, evince the influence of the knowledge thus attained on his mind. We believe that the lecturer had alluded to the fact that persons attempting suicide usually cut across the *front* of the neck, and that the wound thus inflicted is not necessarily or speedily fatal; whilst any one who was acquainted with the anatomy of the parts would make his incision on the *side* of the neck, over the carotid artery and jugular vein. This very course was

* See Cormack's Thesis, chapter iv.

† See Edinburgh Med. and Surg. Journal, vol. xlix. p. 209, &c.

adopted in a most extraordinary manner by the subject of the present relation.*

Two wounds were inflicted by Mr. Doherty, one on each side of his neck, running nearly parallel with the ramus of the lower jaw. On the left side, we learn from Dr. Handyside that "the wound extended obliquely downwards from the lobe of the ear towards the junction of the body with the left cornu of the os hyoides, measuring two inches and a half in length; and it appeared from its direction to have been made with the instrument held in the left hand of the individual. [It was found upon enquiry that Mr. D. was ambidexter.] The platysma myoides to the breadth of two inches and a half, the anterior two thirds of the sterno-mastoid, with the trunk of the spinal accessory nerve, and the lower part of the parotid gland, had been freely divided. Of the parts beneath, the conjoined origin of the occipital and posterior auris arteries, with the posterior facial vein, were found cut across; and through the latter of these vessels, several globules of atmospheric air were found to have entered the internal jugular vein."

The wound on the right side was of very remarkable character. It was four inches in length, the middle part corresponding with the angle of the jaw, but half an inch beneath it. One superficial and two deep wounds had evidently been inflicted, if not by another person, with the right hand of the deceased himself, as was evident from the appearance of the edges of the incision. This was a remarkable circumstance, a suicidal wound on the neck made with the *right* hand being almost invariably on the *left* side; but in the present instance no doubt of its character could be felt. It was found to have laid freely bare the transverse process of the atlas, and to have opened up the space which intervenes between it and the transverse process of the axis, sufficiently to expose to view the vertebral artery. Amongst the parts which were divided were the internal jugular vein with the pneumogastric and anterior branches of the descendens noni nerves; but the carotid artery was itself unwounded. This curious escape appear to have been due to the nature of the instrument with which the wound was inflicted, and the mode in which it had been employed. The razor had a thick blunt extremity, and appeared to have been *dug* into the wound in such a manner that

* Instances of this kind are by no means of unfrequent occurrence. We related in our last Number, p. 171, a case of this kind from Sir Charles Bell; and a parallel one is related by Dr. Graves. "A very remarkable case occurred while I was at Hamburg. My friend, Dr. Oppenheim, was called out one day to visit a man who had cut his throat. The carotids escaped, but the injury inflicted was so great that the patient died after protracted sufferings. His body was given to Dr. O. for dissection. The man who took care of the place happened to be in the room at the time, and Dr. O. said to him in a joking way 'John, have you any fancy to cut your throat? If you have, don't do it in such a bungling way as this man; a little more to the left here, and you will cut the carotid artery.' The individual to whom this observation was made was a very sober steady man, with a family and comfortable subsistence; he never manifested the slightest tendency to suicide, and had no motive to commit it. Yet, strange to say, the sight of the corpse of a suicide, and an observation uttered in jest, made a powerful impression on him; the idea of self-destruction seized upon his mind, and, about an hour after Dr. Oppenheim left the dissecting room, he was horrified at hearing that John had cut his throat. Fortunately he had not profited by the anatomical instructions given to him; he did not cut the carotid, and consequently recovered.—*Lectures on the Institutes of Medicine in Lond. Med. and Surg. Journ.*; vol. vii. p. 69.

whilst the terminal part of its edge divided the sheath of the vessels, and the vein and nerves, this portion had pushed the artery before it. These facts are all interesting when the case is viewed in reference to forensic medicine, as they tend to establish the *intent* with which the wound was inflicted. The following are the details relating to the injection of the vessels with air.

"On examining with great care the contents of the blood-vessels, I ascertained that the internal jugular veins, though empty of blood, were slightly distended with air. The arteries of the neck generally were empty, with the exception of the left carotid, which contained a considerable quantity of air. The middle and inferior thyroid veins, the venæ anonymæ, the descending vena cava and the vena azygos were found partially distended with air, besides containing a little fluid blood. . . . The ascending vena cava contained a little uncoagulated blood and air. On cutting into the liver under the surface of water, air was observed to escape from the hepatic veins; and on treating the spleen and kidneys in a similar way, like results were obtained. The pancreas, and the coronary veins of the stomach presented also the same results. The abdominal aorta on being carefully insulated for three inches in extent by ligatures applied above and below, and also to its branches, and then placed under the surface of water, gave vent on being opened to several globules of air. The femoral, popliteal, and brachial arteries, upon being similarly treated, were observed to contain air."

Now, were this all our information respecting the appearances observed on the examination of this curious case, we should be disposed to regard Dr. Handyside as correct in attributing death to the entrance of air into the veins; and, in fact, this view was taken by Dr. Cormack in his prize thesis, being founded on an imperfect account of it. But Dr. Handyside farther tells us that "on opening the pericardium, the cavities of the heart were found collapsed and nearly empty. The right auricle was perfectly empty of blood with the exception of a minute coagulum at the foramen ovale. This cavity contained some atmospheric air. The pulmonary artery and its minute ramifications contained a very little frothy blood, quite insufficient to separate their walls which were collapsed." Now we have already seen that it was the constant result of the experiments of Nysten, as well as of the later researches of Amussat and others, that whether air was injected into the veins or allowed to enter them spontaneously, in all cases where death took place speedily, the right cavities of the heart and the pulmonary artery were found distended with frothy blood. On this subject Dr. Cormack remarks, as the result of his own experience, that

"If the right auricle be very rapidly distended to such an extent as instantaneously to arrest its contractions, the blood and air are found unmixed; but this is a result which can only be obtained when a wide tube is used, and a great force employed in the introduction of the air. The fact, however, connected with the frothing of the blood, which it is of essential importance for us to bear in mind at present is this: *When an animal is speedily killed by the introduction of air, and bubbles of this fluid are found in the vessels, the right side of the heart is always distended with frothy blood.* Of the correctness of this statement I am perfectly satisfied from the results of numerous experiments, and it is an opinion amply corroborated by those previously performed by Nysten, Magendie, and many others." . . . "It is maintained, then, that the simple circumstance of *the heart being found collapsed, and destitute of frothy blood, of itself affords sufficient proof that Mr. Doherty's death has been erroneously attributed to the air found in the blood-vessels after death.*" (*Remarks*, pp. 7-8.)

Various explanations may be given of the existence of air in the vessels in this case, as well as of the immediate cause of death. Air may have been generated during life, as in the instances mentioned by Dr. Cormack in chap. iv. of his thesis. It appears, too, highly probable that in the case of Mr. Doherty, a considerable proportion of the air entered the vessels after death to supply the vacuum created by the loss of blood, and to fill up the void in the arterial system occasioned by the passage of its fluid contents into the veins, and the subsequent dilatation of their caliber. As there were several arteries as well as veins divided in the case of Mr. Doherty, there can be little doubt that through these apertures a good deal of air must have gained admission after death. As to the immediate cause of death, we think that it has been satisfactorily shown by Dr. Cormack that the quantity of blood lost was probably greater than the pound and a half at which it was estimated by Dr. Handyside; but that the rapid loss even of this quantity was not unlikely to have produced fatal syncope, especially in a person labouring under delirium tremens, as Mr. D. was known to have been, although the circumstance is not hinted at by Dr. H.

It is scarcely necessary for us to go over the arguments adduced by Dr. Cormack in refutation of the doctrines stated by Dr. Handyside on the proximate causes of death from entrance of air into the veins; since we have already given our own opinions on this subject, which nearly correspond with those of Dr. C. We must say, however, that we cannot regard this portion of Dr. H.'s paper as creditable either to his sagacity as a teacher of physiology, or to his candour as a writer. Inferences are drawn which are not in the least warranted by the experiments upon which they profess to be erected; and there is frequently, to say the least, a complete misconception of the meaning of authors from whom he has quoted, and a neglect of prominent facts which militate against his own views. His researches on the subject do not appear to have been very extensive, since his list of authors is evidently taken from Dr. Cormack's thesis; and from mere carelessness he has been led into an error by no means creditable to his discernment. He has quoted Bretonneaux as one of the experimenters on this subject, and has placed him next to Nysten on his list; when the fact really was, as stated by Dr. C. (Thesis, p. 25,) that the *experiments* of this gentleman were on the effects of the injection of pus into the veins, and that in one instance, air was supposed to have entered by *accident*.

In conclusion, we would refer our readers to Dr. Cormack's thesis for many facts and observations which our limits prevent us from alluding to; but must still recommend the question of the introduction of air to the attention of the profession as one which requires to be elucidated by future experiment. The perusal of the report of the Commission will suggest many enquiries which still remain to be made, and furnish many a caution by which the future experimenters may profit. We would urge upon all who may be disposed to prosecute experimental enquiries into this or any other similar subject, the necessity of much previous reflection, of a matured plan, and of minute and scrupulous care in performing every part of their experiments. Let every point which can be submitted to calculation or expressed in numbers be minutely attended to,—such as the quantity of the air introduced, the duration of the experiment, and of its effects, the size and strength of the animal,—let all the conditions of all the experiments, excepting always the point to be ascertained, remain the same, and the same certainty may be obtained with regard to the phenomena of life which already characterizes much of our knowledge of the laws which preside over dead matter. We are convinced that the uncertainty of our knowledge, on many points of importance, depends more on the defective methods we employ than on the essential difficulties which beset our investigations.

---

